# Occupational Health and Safety Vulnerability of Recent Immigrants and Refugees

**DOI:** 10.3390/ijerph15092004

**Published:** 2018-09-14

**Authors:** Basak Yanar, Agnieszka Kosny, Peter M. Smith

**Affiliations:** 1 Institute for Work & Health, 481 University Ave., Suite 800, Toronto, ON M5G 2E9, Canada; akosny@iwh.on.ca (A.K.); psmith@iwh.on.ca (P.M.S.); 2 Dalla Lana School of Public Health, University of Toronto, 155 College Street, 6th floor, Toronto, ON M5T 3M7, Canada; 3 Department of Epidemiology and Preventive Medicine, Monash University, 553 St Kilda Road, Melbourne, VIC 3004, Australia

**Keywords:** occupational health and safety vulnerability, immigrants, employment, qualitative

## Abstract

Recent immigrants and refugees have higher rates of work-related injuries and illnesses compared to Canadian-born workers. As a result, they are often labelled as vulnerable workers. This study explored the factors that contribute to occupational health and safety (OHS) vulnerability of recent immigrants and refugees with a focus on modifiable factors such as exposure to hazards and access to workplace protections, awareness of OHS and worker rights, and empowerment to act on those rights. Eighteen focus groups were conducted with 110 recent immigrants and refugees about their experiences looking for work and in their first jobs in Canada. A thematic content analysis was used to organize the data and to identify and report themes. The jobs described by participants typically involved poor working conditions and exposure to hazards without adequate workplace protections. Most participants had limited knowledge of OHS and employment rights and tended to not voice safety concerns to employers. Understanding OHS vulnerability from the lens of workplace context can help identify modifiable conditions that affect the risk of injury and illness among recent immigrants and refugees. Safe work integration depends on providing these workers with information about their rights, adequate job training, and opportunities for participating in injury prevention.

## 1. Introduction

Immigration is a key driver of Canada’s labor market growth, yet newcomers often face challenges that restrict their access to quality employment, such as devaluation of foreign credentials and experience [1,2,3,4], employers’ prejudices [5,6,7], lack of professional networks [8,9] and language barriers [10,11,12]. In light of these challenges, compared to Canadian-born workers, immigrants are less likely to work in jobs that match their education level and professional experience [10,13,14,15] and typically earn lower wages [16,17,18].

The precarious situation of recent immigrants and refugees in the labor market also has important implications for occupational health and safety (OHS). Overqualification has been linked to work injury [19] and lower pay can result in working longer hours, which has also been associated with increased risk of injury [20]. In addition, compared to their non-immigrant counterparts, immigrants are more likely to perform physically demanding jobs [21,22,23], be exposed to OHS hazards [24,25,26,27,28], and lack health and safety protection [29,30,31]. They are also less likely to receive formal job training and information on health and safety [24,32,33]. As a result, immigrants have higher rates of work-related injuries and illnesses compared to non-immigrants [26,34,35,36,37,38].

Due to increased risk of injury, immigrant workers are often labelled as vulnerable workers [39,40]. In defining the vulnerability of immigrant workers, the majority of studies in the OHS literature focus on the migration and socio-demographic factors (e.g., immigration status, race, language) or on the occupation/industries in which they typically work [22,25,26,39,40]. However, workplace context is being increasingly recognized as a modifiable driving factor of OHS vulnerability [41,42,43,44,45]. Moyce and Schenker [41], in their recent review of research on the OHS of migrant workers, suggested that unregulated and unsafe working conditions (e.g., physical hazards, lack of safety practices) play a role in increasing the risk of injuries. Focusing on the workplace context, Smith and colleagues [42] developed an OHS vulnerability framework comprised of four work-related dimensions that increase risk of injury: exposure to workplace hazards, inadequate workplace-level OHS protections and policies, low worker OHS awareness, and/or a culture that discourages worker participation in safety. While none of these dimensions are novel, they specifically posited that OHS vulnerability is created when workers are exposed to hazards in combination with inadequate access to protections to control those hazards.

Workplace hazards are usually defined as working conditions that have potential to cause injury and/or illness to workers. Examples of hazards are exposure to hazardous substances, working with dangerous tools and equipment, or performing potentially injurious tasks such as repetitive movement and heavy lifting [46]. Hazardous work has been linked to work-related injuries [47]. Risks and hazards are also negatively related to worker participation in safety and safety compliance [48]. Workplace-level OHS protections and policies aim to protect workers from exposure to hazards and other workplace risks. Examples of workplace protections are access to proper safety equipment, identification and controlling of hazardous substances and replacement of defective equipment [42]. Safety policies can include organizational polices related to compliance with OHS standards, communication about and responsiveness to health and safety issues, and workplace health and safety training provided to workers [42,46]. OHS awareness refers to individuals’ awareness of safety hazards in the workplace, knowledge of OHS rights and responsibilities and how to perform their job safely (e.g., how to safely use protective equipment) [42]. Safety knowledge can be acquired through workplace trainings and on the job experience [49]. Knowledge of OHS rights and responsibilities and how to perform their job safely can improve workers’ safety and reduce injury risk [50]. OHS empowerment refers to the extent to which individuals feel free to voice health and safety concerns, ask questions about health and safety and refuse unsafe duties [42]. In a review of studies exploring the relationship between workplace factors and workplace injuries, Shannon and colleagues [51] found that worker empowerment was related to lower injury rates.

Smith and colleagues suggested that vulnerability is defined as situations where workers are exposed to hazards in combination with inadequate protections from these hazards (OHS policies and procedures, awareness of rights and responsibilities, and worker empowerment) [42]. Figure 1 demonstrates the OHS vulnerability conceptual framework for how hazards and lack of workplace protections overlap to create vulnerable conditions. Workers who are exposed to hazards in the workplace in combination with inadequate workplace OHS policies and procedures, low awareness of rights and responsibilities, or a workplace culture that discourages worker participation in safety are considered most vulnerable and at greatest risk of occupational injury and illness. OHS vulnerability framework led to the development of a measure to define more accurately which workers are vulnerable to work injury and illness. In a quantitative study comparing these dimensions of OHS vulnerability between recent Canadian immigrants and workers born in Canada, Lay and colleagues [45] observed that recent immigrants were more likely to experience exposure to work hazards, less likely to access to protective policy and procedures in the workplace and were less empowered to participate in injury prevention in their workplace (e.g., through voicing safety concerns).

However, the exact mechanisms that lead to these differences in dimensions of OHS vulnerability between recent immigrants and Canadian-born workers are not fully understood. For example, Lay and colleagues [45] suggested that immigrant workers experience higher levels of policy and procedure vulnerability. Lack of access to workplace protections may be a result of recent immigrants’ precarious work status (e.g., casual work, short contracts), but it could be also due to their lack of awareness of their rights in the host country. Recent immigrants may experience empowerment vulnerability and not raise safety concerns due to fear of reprisal and losing their jobs [52,53], not fully knowing the safety hazards in the workplaces [26], and/or thinking that speaking up will not make a difference in their workplace.

Understanding the experiences of recent immigrants and refugees regarding accessing workplace protections (e.g., protective policies and procedures, OHS awareness and empowerment) can help to identify important levers for change that could help reduce the risk of injury and illness among all workers, but recent immigrants in particular, if acted upon. Using a qualitative approach, this study examined the experiences of recent immigrants and refugees in looking for work and in their first jobs. We mapped these experiences onto the dimensions of the previously proposed OHS vulnerability conceptual framework from Smith and colleagues [42].

## 2. Methods

The findings come from data collected as a part of a larger qualitative study examining the work-integration process of newcomers in Ontario, Canada and determine key training and resource needs and opportunities related to safely integrating into the labor market. The larger study included interviews with policy makers, service providers, and program developers as well as focus groups with recent immigrant and refugees about the scope of current programming and possible approaches to integrating OHS resources into service delivery. This current analysis specifically focuses on the findings of the focus groups that examined recent immigrant and refugees’ experiences looking for work and in their first jobs in Canada. The research protocol was approved by the University of Toronto Ethics Review Board (REB# 33672).

### 2.1. Recruitment and Sampling

Participants were recruited through settlement organizations and community groups that help recent immigrants and refugees in the Greater Toronto Area (GTA), Eastern Ontario, and Northern Ontario. Settlement organizations publicized the study information through their programs and services (e.g., language, employment programs) to help identify potential participants. The program facilitators announced the study and distributed study information to their classrooms. Study information letters were also posted in the reception area of the settlement agencies and community organizations. In addition, key contacts in community groups disseminated study information to their contacts in the community.

Individuals interested in participating in the study were asked to provide their information using a sign-up sheet or directly contact the researchers if they chose to. Arabic-speaking peer researchers translated the recruitment materials for Arabic focus groups, the largest language group in our sample. All potential participants received a study information letter explaining the details of the study and privacy and confidentiality information. Participant recruitment continued until a diversity of participants was achieved (e.g., immigration stream, gender, professional background) and continued until no new themes were forthcoming in the data.

### 2.2. Procedure

Eighteen focus groups were conducted between February and May 2017 with recent immigrants and refugees who were looking for work or who have had work experience in Canada. The purpose of the focus groups was to understand the issues newcomers face as they look for and start employment in their first jobs. Focus group questions focused on the employment preparation process, what kind of work-related resources and support they accessed, their knowledge of rights related to employment and safety at work as well as their experiences in their first jobs.

Four Arabic-speaking peer researchers were recruited to facilitate focus groups with Arabic-speaking participants. Peer researchers were trained by researchers in ethics, recruitment, and data collection. They were also provided study-specific training regarding focus group administration.

All focus groups took place in a private room in community organizations. Each focus group included approximately four to ten participants and lasted between 60–90 min. All participants signed a consent form acknowledging their willingness to participate in the focus group, and their awareness that the focus groups will be recorded and transcribed. Participants received a $20 gift card as honorarium.

### 2.3. Participants

In total, 110 newcomers participated in 18 focus groups (13 in English and 5 in Arabic). Participants entered Canada through different streams: out of 92 participants who reported their immigration stream, 25% were economic immigrants which also included spouses of principal applicants, 35% were humanitarian refugees and 9% belonged to family category. 15% had temporary status. Majority of the participants (70%) had been in Canada less than 3 years, 17% reported being in Canada between 3–5 years.

Participants were from a range of geographic regions such as South Asia, South America and the Caribbean, and Europe with just under half being from Middle East (54%). Close to half (43%) of the participants were under 35 years old, followed by 29% between 35–44 years, 17% between 44–56 years, and 9% older than 56 years old. Fifty-five percent were women. Majority of the participants (73%) had university training. They belonged to a variety of professions before coming to Canada which included engineering (12%), education (22%), skilled trade (12%), business (11%), and health (5%). Thirty-one percent of the participants were employed at the time of the focus groups; however, most participants reported having had some kind of work experience (e.g., volunteering, short contracts, casual work).

### 2.4. Data Analysis

All focus groups were audio recorded and transcribed. Peer researchers who facilitated Arabic focus groups translated and transcribed these focus groups. A research team member with proficiency in Arabic read the translated transcripts to ensure fidelity between the recordings and transcription. Transcripts were entered NVivo (qualitative data analysis software, QSR International Pty Ltd., Version 10) for data storage and organization [54].

Thematic analysis [55] was used to organize the data, and to identify and interpret themes. Three researchers read a sample of focus group transcript to highlight key themes and phrases and establish a preliminary list of codes. After the preliminary coding list was discussed among the research team, a coding manual was developed. The coding manual consisted of definition for each code with an explanation of how this code would apply to the research objectives. Transcripts were then coded in two rounds by two researchers. Once a researcher completed the first round of coding, the coded text was then sent to the second researcher to add additional codes or to identify codes that may have been miscoded. The content assigned to the codes was reviewed by the researchers to identify themes, patterns, gaps and contradictions in data. Common themes and concepts across codes that captured key insights were identified. Constant comparison method [56] was used to explore the relationship between the themes emerging from each focus group and to assess similarities and differences between these themes. Any discrepancies in coding and interpretative differences were discussed in team meetings and resolved through discussion until full consensus was reached. A second analysis was carried out to organize and map themes onto the dimensions of OHS vulnerability: hazard exposure, OHS policies and procedures, OHS awareness and OHS empowerment. In reporting the findings, some minor details were removed from the quotes in order to preserve participant anonymity. Where possible we have included occupational or industrial information to contextualize quotes, however, given the nature of focus group discussions this was not always possible.

## 3. Findings

### 3.1. Hazardous/Dangerous Work

All participants reported experiencing challenges with finding employment in Canada due to employers requiring Canadian work experience, lack of recognition of their credentials, and language difficulties. Sending out resumes yielded very little success for most participants. Many found their first jobs through their community and ethnic connections (e.g., friends, family, and connections from their cultural/religious community). Some participants also found their first jobs through temporary work agencies and settlement agencies which connected them with employers. Although study participants came from diverse professional backgrounds, their first jobs in Canada were mainly concentrated in manufacturing, trade, construction, and customer service.

First jobs were typically casual, short term, and low wage contracts and in temporary agencies and small businesses run by other newcomers. Although these jobs were easier to find because they did not require high language proficiency and Canadian experience, participants were exposed to poor and dangerous working conditions. For example, participants talked about working in a packaging/assembly line at a high pace, working in the cold for long hours without proper equipment and adequate breaks, lifting heavy loads, and working with chemicals. These jobs were described by participants as undermining of their skills and experience and physically exhausting.

Most participants who were performing manual labor or physically demanding work did not have any previous experience with this kind of work before and found themselves in these new work conditions with little knowledge of performing these tasks or using the tools and the machinery.

I’m new, and I don’t work in the laborer. I’ve been working in computer in my country. It’s different from … I have experience to different [work], and when I come, people is not perfect and when they are do that, do that, okay, I will do it. And one day, I’m [working] in the big cold fridge

*(Participant currently working in a manufacturing plant (), Focus Group 13, GTA)*

Some participants were working in jobs that were not physically demanding; however, they faced other kinds of hazards or dangerous situations. For example, one participant who took a job at a convenience store as a cashier had to work night shifts dealing with “drug users at night” without any safety training and had to learn how to deal with these dangerous situations themselves:

R (R: respondent, M: focus group moderator): In the first job, never [received safety training]. I know that through my work, never, no safety, no health and safety procedures. Just I know how many hours I would work and (inaudible) That’s it … I understand that through the problems that happens in the stores with me because I was working on the night shifts and what happened when I was there. So, I know after that how to call the police and how to call deal with that.

M: Would you mind sharing with us what happened that night?

R: They came to me, masked people with guns in their hand and they took all the cash that I have. I opened it actually. But they don’t touch me. Just open the cash, open the cash, like this and they threatened, and I opened everything and they left.

*(Participant working in a convenience store, Focus Group 11, GTA)*

Few participants also described being mistreated such as being yelled at or threatened by their employers. There was also some mention of discrimination experienced by participants and their vulnerable immigration status (having a social insurance number that starts with 9 which indicated temporary status) being a factor in precarious work situation:

R: That’s how they begin abusing you. They overwork you, because knowing you have a SIN [social insurance number] number with a nine, that means you’re new. It’s the first thing that shows that you’re new. They’ll overwork you, they’ll give you less pay, they’ll mistreat you at the job, because they know you need the job and it’s hard for newcomers to get a job in Canada.

*(Focus Group 11, GTA)*

### 3.2. Workplace Protections and Procedures Related to OHS

Although most participants were working in first jobs that they did not have experience in, only a small number reported being provided with job-related training or formal job instruction. Training typically included minimal or no information on health and safety. This was especially true for jobs found through temporary employment agencies and small businesses in participants’ own community. Most participants were left to learn these tasks on their own.

R: No. I didn’t [receive safety training]. I started right away, despite the fact that it was my first time working in this field of work.

M: You never worked in a similar job before? And there was no training, you know, a couple days maybe, they teach you how to do the job?

R: No, nothing. I went straight into the work.

*(Participant currently working in a supermarket, Focus Group 18, GTA)*

Some participants talked about being given instructions by an experienced co-worker or a supervisor after they started on the job. In some instances, workers talked about having trouble understanding and following their supervisor’s instructions due to language difficulties. These individuals felt that they had the responsibility to make sure they understood the instructions and performed the job safely.

There were examples of workers performing jobs and using tools without protection and being asked to carry heavy loads without proper equipment. Only few participants reported being provided with proper protective equipment (PPEs) by their employers. One example of not being provided with PPEs occurred when a worker—who was not used to working in cold conditions—was asked to move metal equipment in a construction area. This job took hours and the worker was not provided with adequate glove wear:

It was in the winter. It was 11 degrees below zero, but the wind-chill made it 19 below and we were working outside. I didn’t have the right gloves, so my hands were freezing. I mean, not that I felt like they were freezing, I mean they were freezing. I went on the opposite side of the road on our break-time and went to where I knew there was a public laundry. I went in and put my hands on the dryer. It took about 15 min for my hands to thaw out and the blood to circulate again.

*(Participant working in a construction site, Focus Group 11, GTA)*

The worker felt that that it was his own responsibility, not the employers, to make sure he had PPEs. When asked whether he requested the right gloves from their employer, the worker shared:

No. I didn’t ask them, either. I think that was my responsibility, to get the right glove. But I’m just saying, because of not being familiar with the conditions, it took me a while. Not being familiar with what’s needed, it took me a while. I spent a lot of time trying to get the right gloves, but you want to see if you can minimize your expense, you don’t want to go and just buy the most expensive glove, you’re trying to get a good glove and see if you cannot pay an arm and a leg for it. That takes a lot of time. You have to search and get. That’s where I was at the time, trying to get the gloves to handle what had become extreme conditions.

*(Participant working in a construction site, Focus Group 11, GTA)*

There were other examples of workers buying their own PPEs to protect themselves from hazards at work such as chemicals and dust. In a few instances, workers reported that employers reacted negatively by reprimanding them for using their own PPE. One participant shared that she started wearing a mask as she was allergic to a substance in the workplace and consequently was moved to another unit by a supervisor to prevent other workers following suit.

No masks, so I bought a mask by myself but they didn’t like me to put it on, because it’s mean here, not safety, not good for everyone. But I know changing me to the other section, I was quiet, I didn’t want to say anything

*(Participant currently working in a manufacturing plant, Focus Group 6, GTA)*

Overall, the discussions in the focus groups suggest that participants were working in jobs that were new to them without proper safety practices and protective equipment. In workplaces, there were few practices and policies in place to protect workers from exposure to hazards (e.g., chemical liquid, heavy loads). Also, it was clear that unsafe practices were common in some of the workplaces, which made it difficult for workers to challenge.

You do it because you see even your employer doing it. And because he is experienced, he can do it. I was not, so I burned myself. So, in general ideally, you’re supposed to report to your supervisor, but when the supervisor himself is doing it, who are you going to report it to? That is the trouble in small businesses. Maybe in a big one you will try and get something done.

*(Participant working in a pizza shop, Focus Group 4, Eastern Ontario)*

### 3.3. OHS Awareness

Most participants in our study had participated in some language and employment preparation programming through settlement agencies and community organizations, which typically focused on job search and resume building and cultural competency training. Many participants reported not receiving information about employment standards or their OHS rights in these programs. A minority had attended programs which introduced them to some employment standards and health and safety information. However, these were often “one-off” workshops rather than systematic programs.

Only a small number of participants reported receiving OHS related training in their workplaces, such as being given handouts or completing an online training, and in few cases receiving in-person training. Many participants knew very little about the responsibility of their employer vis-a-vis training and safety policies and procedures. For example, when asked about whether they received information on how to work safely or what to do when injured, they reported not receiving this information from their employers. The quote below—from a participant formerly worked in an industrial bakery is reflective of many conversations arose in other focus groups:

M: When you start a job, they give you information. Did anyone tell you, if somebody treats you badly, these are the things that you should do?

R: No. Nothing.

M: So, you just ignored it.

R: Yeah.

*(Participant working in an industrial bakery, Focus Group 12, GTA)*

Participants who had some knowledge of their worker rights reported learning about these rights from their co-workers. They relied on and trusted other workers to guide them in learning how to be careful and what to avoid and perform their job safely.

Most of the times we know our rights from the co-workers. Because this is a new place, so we don’t know anything about the work culture and anything. When we started the work, most of the rights, we can know the responsibilities, but rights, most of the co-workers this is our rights so we can do like this. If we have any problems, when we discuss them, they say, oh, yeah, we can do like that. Because senior co-workers, full-time employees, most of them in my case I know from them.

*(Focus Group 5, Northern Ontario)*

One theme that emerged from the data was the degree of confusion regarding participants’ understanding of OHS concepts. For example, when asked about their knowledge regarding their rights at work, some participants responded by listing their responsibilities in the workplace, such as the work tasks they were expected to complete in their jobs. In some instances, participants simply described signing a contract that outlined their responsibilities or required to take a quiz by the temporary agency without receiving training before being assigned to a job.

R: [Temp agency] gave a form and they had questions there about what should we do if in any case, and there were options. For example, wear safety shoes and for example a sign of a fire, and all that, but they didn’t inform me about that, they just took an exam.

M: It was a quiz?

R: Yeah, it was kind of a quiz.

M: But they didn’t give you that information beforehand.

R: No.

*(Focus Group 6, GTA)*

Also, when asked about their rights, some participants talked about employment rights information from their native country and thought these applied to Canadian workplaces as well:

R: For me, it’s just the general work rules and regulations that I’ve had from back home…. I imagine it would be the same thing. Of course, they give you three months, or whatever, for sick leave. It’s just the general idea that we have … You know, the common-sense things.

*(Focus Group 10, GTA)*

At times, workplace health and safety were understood to be the measures taken to protect clients and customers, not the workers themselves. For example, when a participant who had received injury prevention training was asked to elaborate on this training, it was clear that the training was intended only for the safety of the elderly clients:

M: What about you in [healthcare sector], anything around what to do if you were injured?

R: I have experience about I worked there. They told me we have training about if the seniors fall, but for me first I think to help, but the training if you can help, you help. But if you can’t, you need to phone the ambulance to come to help.

M: That’s more for your clients. Anything about you though, if you got injured? Let’s say you were helping a client and you were injured, is there anything about what to do if you were injured?

R: I don’t know.

*(Participant working in healthcare sector, Focus Group 5, Northern Ontario)*

When describing their jobs, it was apparent that participants were performing unsafe tasks without being aware of the safety risks or that protections were missing. For example, participants were talking about lifting heavy weights but not aware of the safety hazards of these. Also, some participants mentioned that they had learned about their rights, and the responsibilities of their employer, only after they left their jobs instead of while they were still working.

Overall, most participants had very limited knowledge about employment standards and employers’ OHS responsibilities. Most participants did not receive OHS training, this was especially true for participants working in small workplaces. As mentioned earlier, only a handful of participants reported receiving OHS training in their workplaces. These participants were given information about the hazards in the workplace, their rights and were receiving ongoing training. One participant described his experience as positive:

Yeah, the job that I was previously doing had done nothing, which has resulted in many hazards. But the job I’m doing right now is through a company called [company name], so they had given a proper training. They give you training before. They train when I started the job and there is an ongoing job training too, which when I’m doing something wrong, they keep on reminding me that this is the way you do it. This job has been great, but the previous experience is really bad

*(Focus Group 4, Eastern Ontario)*

### 3.4. OHS Empowerment

Although many participants reported working in precarious or unsafe situations, most reported not speaking up to their employers about hazards and injuries or were not sure about what to do when asked to do something unsafe. When asked about the reason for not speaking up, participants reported that they were worried that speaking up would result in being fired, or in the case of temporary agency/casual workers, not being rehired when their contracts were finished. Some participants felt that they had to stay in precarious jobs to get Canadian work experience as many gave up hope that they would find a better job. Few also mentioned that they needed to endure these situations until they got their credentials approved or started re-education in Canada. Other participants shared that they needed to keep their jobs to support their family in Canada or in their home country.

Because we don’t want to say because we don’t want to make trouble also for ourselves too, because if we make too much trouble with this company, maybe it will be hard to be hired at some other company. This is one thing, we are newcomers, we’re scared. And the other thing for hurt, it means it’s a dangerous thing, the company never want you to say, never to say it’s a kind of danger for them.

*(Focus Group 6, GTA)*

Some participants talked about language difficulties or being new to their jobs creating a situation where they could not speak up but felt that once when they become familiar with the work environment and labor market, then they would be more comfortable voicing their concerns.

In my initial period, I’m ready to compromise these types of things because my main thing is just to get and stay in a job for a period of time. If I am familiar with all the things, all the Canadian surrounding things, then I’ll be ready to speak up against.

*(Focus Group 6, GTA)*

However, speaking up did not always work out. A few participants complained about unsafe working conditions or told their employer about an injury only to be reprimanded. There were examples of power dynamics at play, and examples of workers being dismissed upon objecting to something they deemed unfair. One participant shared that he was told by the recruiter at a temporary work agency that if he complained about his injury, his complaint would go on his file and no one would hire him again.

In a few instances, when the workers hurt themselves they were told by their employers not to say anything.

I’m working at the freezer and I stay for eight hours, no giving us a break ... I feel sick one day, sick like I can’t breathe, and the cold... one day I passed out, don’t feel good and no sitting [in the] room, nothing ... Somebody came in and tell me, you’d better not say words [...] You’re not allowed to talk, you’re not allowed to talk. You talk to the next person, we change you for another department.

*(Participant working in a food processing plant, Focus group 13, GTA)*

While in some instances participants mentioned that the employer/supervisor witnessed the injury, there was no follow-up by the employers. For example, upon complaining about hazards, a worker was moved to a different department and the hazard was not addressed. In another instance, a worker was told by their employer that it was okay to speak up and ask about their rights, but nothing changed in employers’ practices.

When I was working at the restaurant, He [the chef] gave me the sheet for the conditions of employment and explained it to me. I expect that you should sit down and speak up and ask all the questions, but the problem is when I asked him about the breaks—I have been working standing on my feet for 9 hours—He told me there is no breaks. He told me that we have a program [referring to piecemeal work]. If you finish it in two hours then you have seven hours left for you but it’s impossible to finish it in two hours. It’s more realistic that you would finish it in 10 hours so he always gives you less time than what is required.

*(Participant working in a restaurant, Focus Group 18, GTA)*

It was apparent from many conversations that participants did not know that they could report an injury and submit claims to the workers compensation board. After an injury, some participants, not knowing they could report a claim, either continued working with the injury or left their jobs and did not go back. Only in one instance was a claim filed by the worker’s healthcare provider when she went to the hospital following an injury. However, when the employer found out, she was scolded by the manager:

R: They don’t want to say anything. And the first time, what’s happened, what’s going on? I injured my hand. Oh, you tell the doctor I am injured in the workplace? Yes. Why? Because I’m not in my home, it’s the workplace.

M: You just told the truth.

R: Yeah. Because of that, yeah, he [the employer] is not happy for that.

*(Participant working in a food processing plant, Focus Group 13, GTA)*

Participants who found their jobs through community connections or service providers faced the challenge of damaging their personal connections if they spoke up. For some, speaking up against a relative or an elder ran counter to cultural norms. They felt that they should be grateful for the opportunity given and did not want to damage their relationships with their employer/manager. Being bound by these social pressures, they were unlikely to complain. 

Overall, speaking up when asked to do something unsafe or when experiencing mistreatment was difficult for the participants due to some of the circumstances described earlier such as the pressures of keeping a positive relationship with employer and fear of losing jobs. In addition, empowerment vulnerability was not just a result of their precarious situation but also was related to their lack of knowledge about their rights, workers compensation system, and how to report injuries. For example, some workers were not aware of what needs to be done after an injury so they either continued working injured or left their jobs. The quote from the below focus group participant illustrates this challenge:

What I felt the maximum was when we come in and we don’t get a proper job, we try to take up anything that we get. And due to the lack of awareness of our rights, like as a worker we have some rights, there has been a lot of misuse you can call it, like being underpaid, being not given what we are supposed to get. That is the biggest challenge that I feel was there.

*(Focus Group 4, Eastern Ontario)*

## 4. Discussion

This study examined the experiences of recent immigrant and refugees in looking for work and in their first jobs in Canada through the lens of a recently described conceptual model of OHS vulnerability [42]. The findings from this study add to our understanding of modifiable factors faced by recent immigrants and refugees that influence their risk of work injury.

Most participants in this study did not have previous experience related to their first jobs in Canada and were performing physically demanding jobs for the first time. Being new to their jobs and the Canadian work environment contributed to a lack of important safety knowledge. This finding reflects previous research that being new to the host country and to their jobs, recent immigrants may not be fully aware of the safety risks in their jobs and may not have access to information regarding worker and OHS rights [57,58,59]. While talking about their jobs, some participants were not aware that tasks they were performing were hazardous or that they required PPEs to perform such tasks. Past research suggests that the mismatch between education and occupational skill requirements (i.e., overqualification) is associated with risk of work injury [19]. A person with advanced educational and professional experience, when suddenly faced with a manual labor setting, may not adequately understand the hazards present in this type of work environment. When a workplace lacks adequate job and OHS training, and protective policies, newcomers may be at a high risk of injury. Under the Occupational Health and Safety Act, employers have the highest responsibility to keep workers safe at work. Proactive, targeted inspections and audits by regulatory bodies that are focused on industries who hire immigrants could help identify employers who fail to provide adequate safe working conditions and help to address some of the vulnerabilities highlighted in this study due to unsafe working conditions.

Although the province of Ontario introduced mandatory OHS awareness training for workers and supervisors in July 2014, only few participants reported receiving OHS training in their workplaces. Many participants knew little about their rights or what to do in unsafe situations or when injured; only those with previous hazardous workplace experience (e.g., construction) before immigrating to Canada were familiar with workplace safety rights and policies. Many participants also reported not receiving information on worker rights or health and safety while taking language or employment preparation programs. While many settlement resources and programs focus on helping newcomers find work, few provide information on employment rights and responsibilities of the employer [60]. Ensuring that newcomers have access to knowledge and training in OHS should be a priority for policy makers, service agencies, and other stakeholders in the immigration and employment field. These resources can be integrated into the formal settlement curriculum such as language and employment preparation programs. Also, this information could be provided in lower-level language classes, as many newcomers in this study were looking for work shortly after arrival in Canada. In some cases, participants talked about learning about their rights after they left their jobs or being reprimanded for speaking up. These participants reflected on their work experiences and wished they were aware of their rights while employed. Instead, they were let go or they left their jobs. Early access to information could empower workers with the knowledge of their rights in the workplace. This information could also be integrated into pre-arrival immigration programs as well as in government resources such as Welcome to Canada guides for newcomers. 

In many cases, participants reported not being provided with adequate PPEs in their workplaces. A possible explanation for this might be that some of the participants were hired by the business owners who were also newcomers themselves and were thus may not fully aware of their responsibilities regarding OHS or may have a limited understanding of OHS requirements. For example, Gravel and colleagues [29], in a study on the OHS management in small business who employ immigrant workers, found that there tended to be inadequate understanding of health and safety issues and implementation of OHS regulations in these workplaces. The lack of organizational policies in these workplaces can be particularly problematic, especially when workers are not aware of their OHS rights. It was clear that in some workplaces unsafe practices were common among supervisors and workers alike. There is a need to develop interventions to improve first-job safety for newcomers, especially in small businesses, as immigrant workers are increasingly more employed in the small business sector [61]. Safety commitment of management is an important predictor of safety performance [62,63], therefore providing training to business owners that hire newcomers on managing health and safety and implementing incentives to be more involved in prevention strategies can help employers creating safer workplaces. The study findings show that the few participants who were given adequate OHS training and protective equipment by their employers spoken positively about these jobs and may not experience the same vulnerability reported by other workers. Therefore, focusing on workplace awareness and empowerment is critically important. Given that many workers in this study reported not receiving mandatory OHS training in their workplaces, it is important for policy makers and government bodies to understand the system-level barriers to successful implementation of mandatory training or other system-level programs related to OHS. For example, it could be that small business face challenges in accessing adequate information regarding legislative changes related to employment standards and OHS. Recently, the Government of Ontario introduced legislative changes (Bill 148) to improve the working conditions for many Ontario workers, including increases to minimum wage and increased protections for workers. Although this comes as a welcome change, it is also important to understand the barriers to protect workers experiencing vulnerability at the system level, such as addressing the occupational mismatch experienced by many recent immigrants.

Finally, most participants in this study reported that they did not speak up when they saw something unsafe and did not report their injuries. The most commonly mentioned reasons for not speaking up were the fear of losing employment—especially when struggling to support their families in their new country—and fear of reprisal. In addition, when participants found jobs through their personal communities, it was especially difficult for them to speak up as they did not want to damage relationships with their employers. The power imbalance between employers and employees in small organizations is a complex issue [64] and addressing these imbalances in the workplace is without an easy answer.

Lack of OHS and workers’ rights knowledge may also contribute to empowerment vulnerability. Some participants who were injured did not know that they had an avenue to report their injury. Not knowing about their rights to refuse unsafe work and to report concerns to the appropriate authoritative bodies may put newcomers in a vulnerable situation [57]. Research suggests that newcomers face challenges in accessing workers’ compensation and health services after a work-related injury [22,57,65]. Awareness and empowerment vulnerability go hand in hand; if newcomers do not know about their basic rights to report and participate, they will not exercise these rights. It is important to provide this knowledge to newcomers and make these resources readily accessible. For example, if workers understand that they can anonymously report health and safety violations to the Ministry of Labour, they may feel more empowered to do so.

Overall, findings of this study provide insights for mechanisms that contribute to the OHS vulnerability of recent immigrants and refugees. In defining OHS vulnerability framework, Smith and colleagues ([42], p. 235) noted that “two workers may be exposed to the same level of hazard potential, but if one is employed in a workplace with active policies and procedures to control these hazards they would be less vulnerable to workplace injury.” Focusing on workplace context as a driver of OHS vulnerability can help researchers, policymakers, and employers to identify and improve the conditions for the safe employment of recent immigrants and refugees.

### Strengths and Limitations

The focus group discussions led to rich information about the experiences of recent immigrants and refugees and offer a useful framework to understand the modifiable work-related factors that are related to OHS vulnerability. Most participants in this study had participated in some language and employment preparation training through settlement and community organizations, the experiences discussed in this study cannot speak to the experiences of all newcomers. There are many newcomers who do not have access to settlement programs and services, it is important to consider how they connect to their first jobs and access OHS information. It is also important to note that most participants in this study had fairly high levels of education and language skills (excluding some refugee participants) as they were recruited via employment programs that require a certain capacity in English. It is likely that newcomers with lower levels of education and poor English-language skills have more difficulty finding work and understanding their rights or exercising them.

This study explored newcomers’ early labor market experiences in Canada. The vulnerabilities discussed in this study may transform with time as immigrants move forward in their careers. Future research can also explore the pathways that immigrants use in learning about their rights in the workplace over time. For example, with time, newcomers may form new networks and gain knowledge of their rights through different resources (e.g., co-workers). Finally, this study focused on the experiences of recent immigrants and refugees in finding work and in their first jobs; future research can examine the experiences of employers who hire newcomers, such as their needs and challenges hiring and training immigrants.

## 5. Conclusions

The findings of this study suggest that most newcomers experience multiple forms of vulnerability—the jobs described involved poor working conditions and unsafe situations without adequate policies and procedures related to OHS, had limited knowledge of OHS and their rights in the workplace and felt uncomfortable questioning their working conditions or reporting hazards or respond to OHS issues. Safe work integration depends on providing recent immigrant and refugees with information about their rights before they find their first jobs in Canada, providing proper job training and encouragement of participating in injury prevention.

## Figures and Tables

**Figure 1:** Conceptual Framework of OHS Vulnerability.

**Table:** 

		Protections (Policies and Procedures, Awareness, Empowerment)	
		Adequate	Inadequate
Hazards	No	Least Vulnerable	Somewhat Vulnerable
	Yes	Somewhat Vulnerable	Most Vulnerable
